# Supplementary figures and images for: Monocyte-eosinophil signaling axis promotes vaccine-mediated protection against SARS-CoV-2
Source: PLoS Pathog. 2025 Dec 2;21(12):e1013752. doi: 10.1371/journal.ppat.1013752 (PMC12688131; doi:10.1371/journal.ppat.1013752)

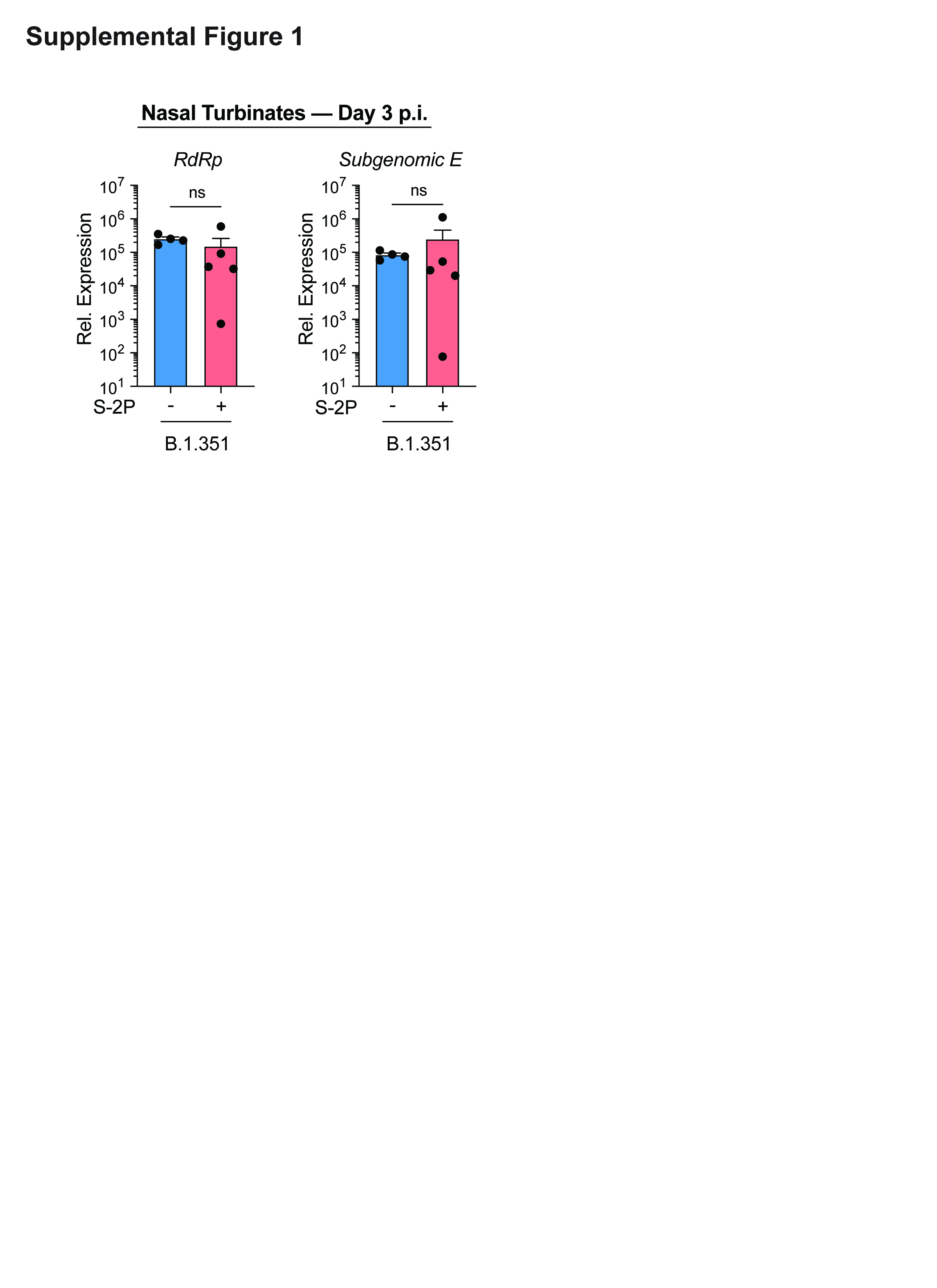

Supplement: S1 Fig — SARS-COV-2 viral loads in the nasal turbinates on day 3 p.i. as measured by RdRp genomic RNA by RT-qPCR (left) and sG E gene RNA by RT-qPCR (right). Group color are as follows: naïve infected = blue, 0.5 µg S-2P vaccinated infected = red. Data are represented by the mean + /- the standard error of the mean. Statistical significance was determined using an unpaired Student’s t test. (TIF) [file ppat.1013752.s001.tif]

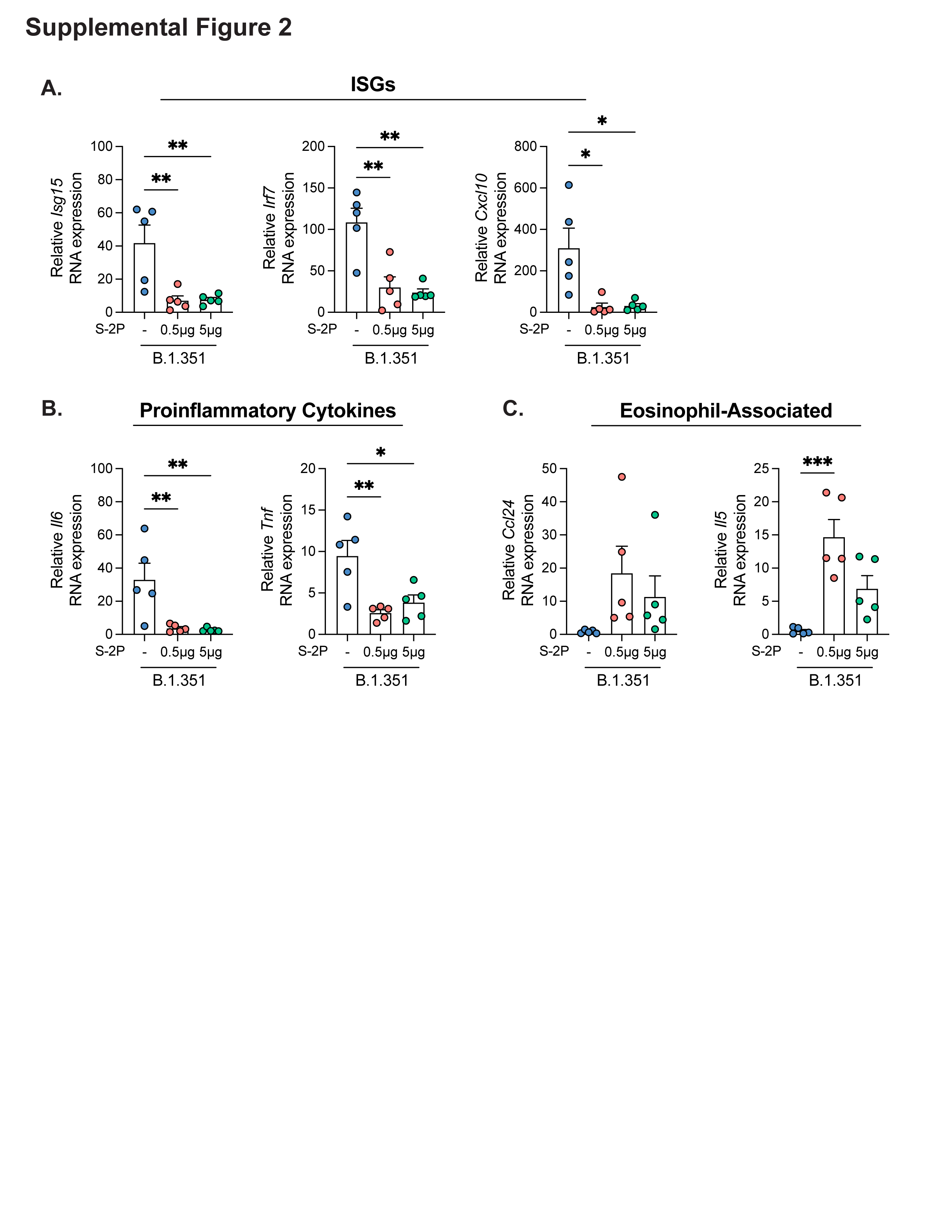

Supplement: S2 Fig — Measured by RT-qPCR. Group names and color are as follows: naïve infected/SARS-CoV-2, blue; low-dose vaccinated infected/0.5 μg S-2P, SARS-CoV-2, red; high-dose vaccinated infected/5 µg S-2P, SARS-CoV-2, green. Data are represented by the mean + /- the standard error of the mean. Statistical significance was determined using an unpaired one-way ANOVA with Tukey’s multiple comparisons test, and P values are represented above the bar graphs as follows: *, P < 0.05; **, P < 0.01; ***, P < 0.001. (TIF) [file ppat.1013752.s002.tif]

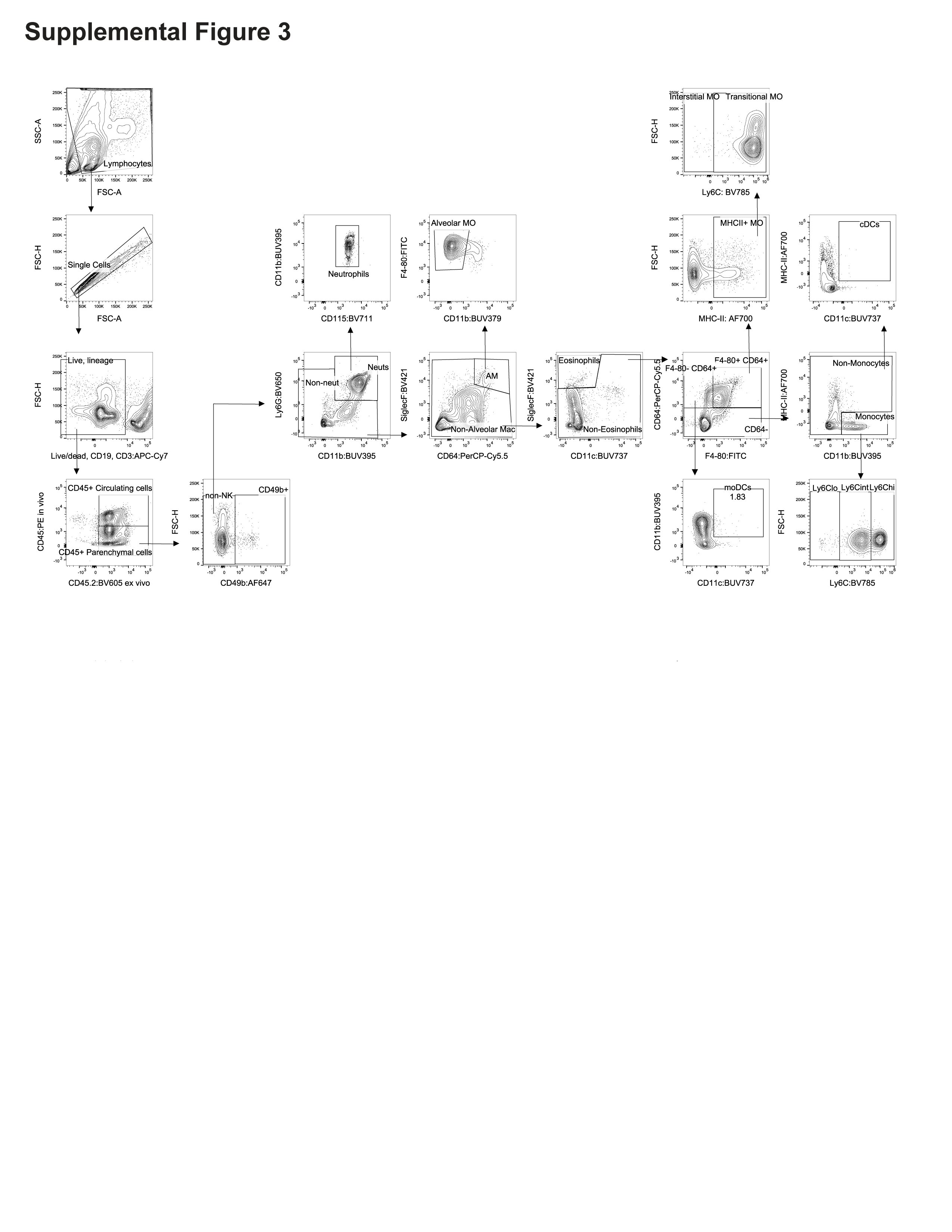

Supplement: S3 Fig — Mice infected with SARS-CoV-2, and lung tissue was harvested on day 3 p.i. Five minutes prior to euthanasia, CD45:PE was injected into mice via the retro-orbital route. Lungs were processed to a single-cell suspension and analyzed via flow cytometry. Cell populations were identified by sequential gating following the black arrows. (TIF) [file ppat.1013752.s003.tif]

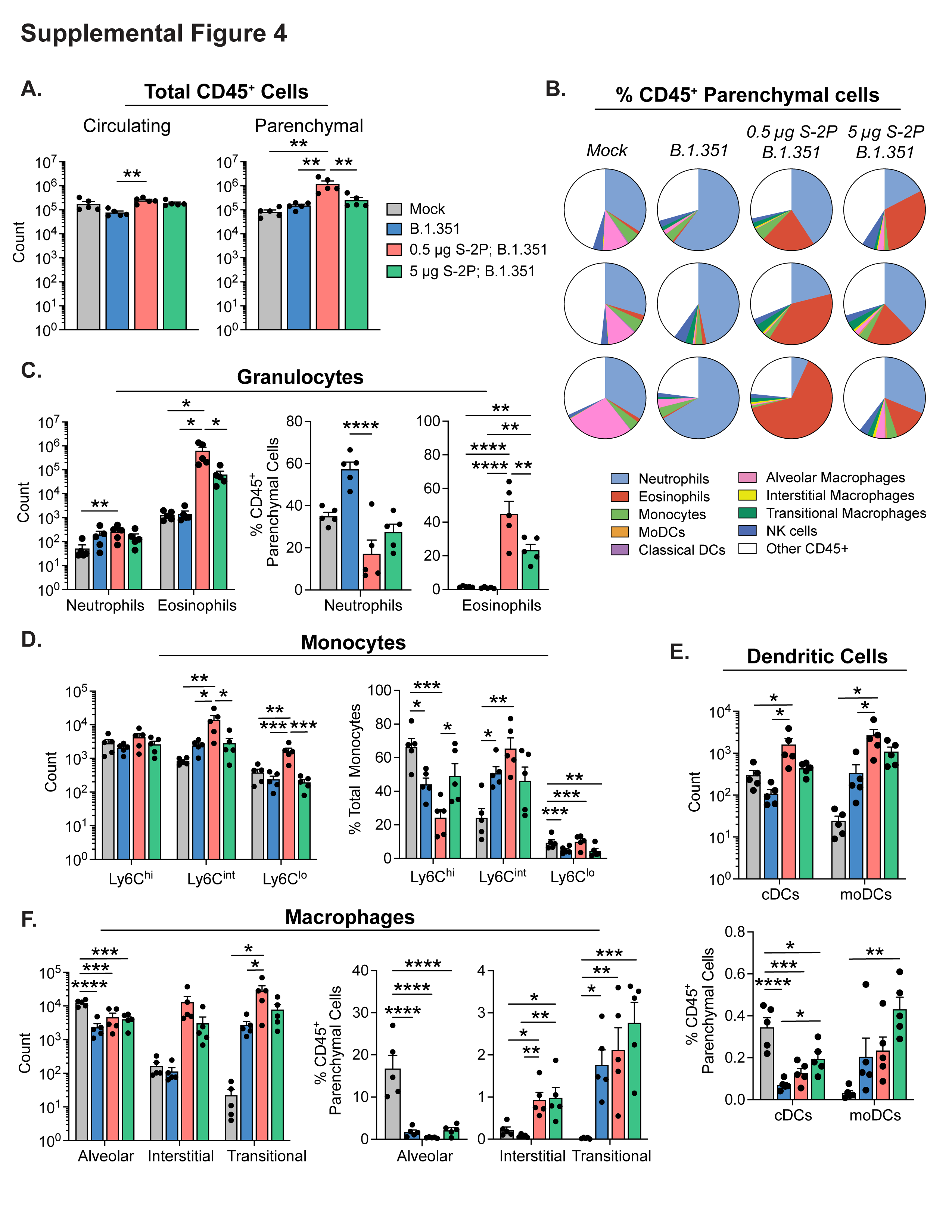

Supplement: S4 Fig — S-2P vaccinated or naïve (unvaccinated) mice were challenged intranasally with 1 x 10^6^ PFU B.1.351 and parenchymal lung immune cells evaluated by flow cytometry on day 3 p.i. (A) Absolute counts of CD45+ circulating cells (left) and absolute counts of CD45+ lung-resident parenchymal cells (right). (B) Pie charts displaying proportion of each identified cell type represented as a percent of the total CD45+ lung parenchymal cells for 3 representative mice from each group. (C) Quantification of granulocytes identified as neutrophils and eosinophils represented as absolute counts (left) and proportion of total CD45+ parenchymal cells (right). (D) Quantification of monocyte subtypes based on Ly6C expression represented as absolute counts (left) and proportion of total monocytes (right). (E) Quantification of classical dendritic cells and monocyte-derived dendritic cells represented as absolute counts (top) and proportion of total CD45+ parenchymal cells (bottom). (F) Quantification of macrophage subtypes identified as alveolar, interstitial, and transitional macrophages represented as absolute counts (left) and proportion of total CD45+ parenchymal cells (right). Group names and color are as follows: uninfected/mock, grey; naïve infected/SARS-CoV-2, blue; low-dose vaccinated infected/0.5 μg S-2P, SARS-CoV-2, red; high-dose vaccinated infected/5 µg S-2P, SARS-CoV-2, green. Data are represented by the mean + /- the standard error of the mean. Statistical significance was determined using an unpaired one-way ANOVA with Tukey’s multiple comparisons test, and P values are represented above the bar graphs as follows: *, P < 0.05; **, P < 0.01; ***, P < 0.001; ****, P < 0.0001. (TIF) [file ppat.1013752.s004.tif]

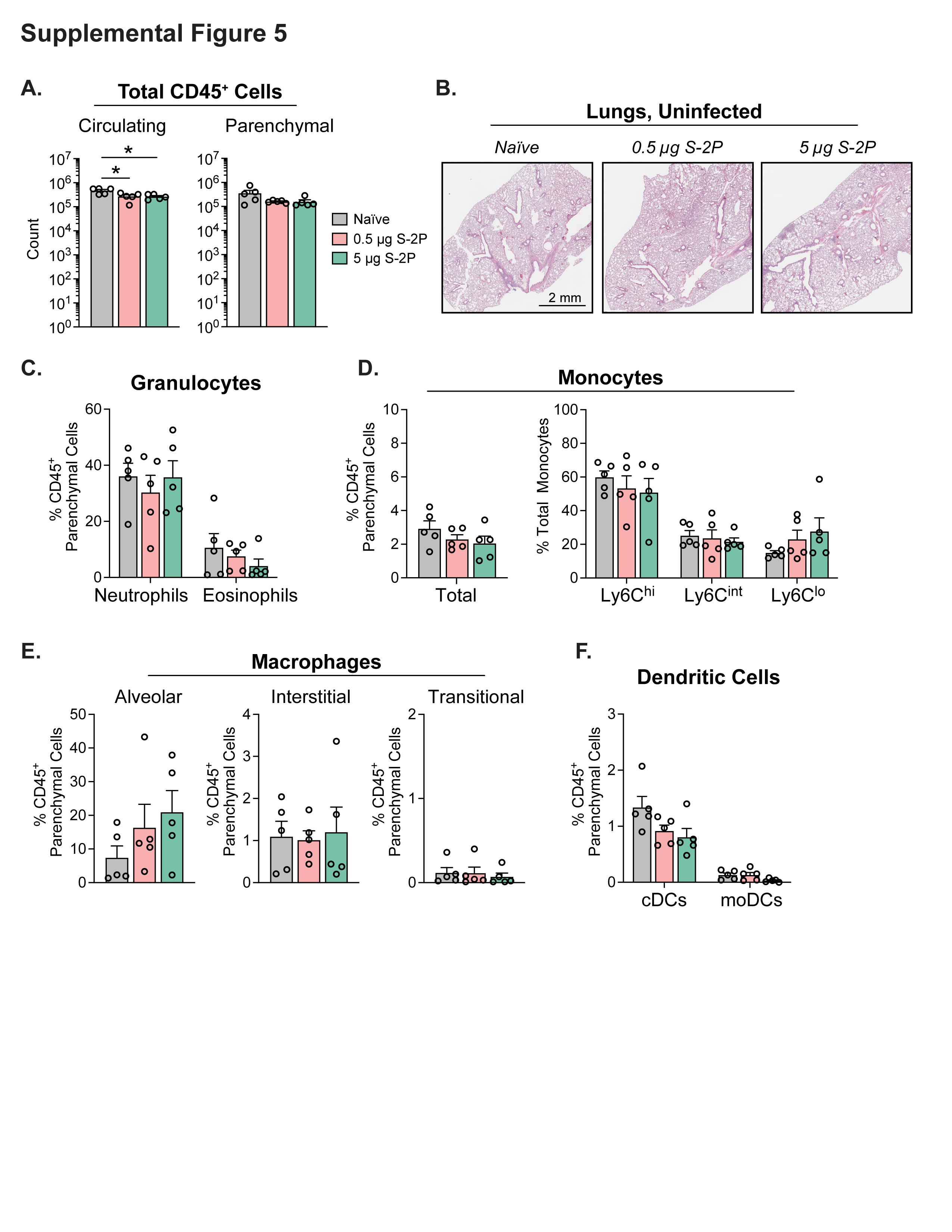

Supplement: S5 Fig — Analysis of lung parenchymal immune cells in uninfected S-2P vaccinated and naïve mice. (A) Absolute counts of CD45+ circulating cells (left) and absolute counts of CD45+ lung-resident parenchymal cells (right) determined by flow cytometry. (B) Histophathologic images of mouse lungs stained with H&E. Scale bars are 2 mm. (C) Quantification of granulocytes identified as neutrophils and eosinophils represented as proportion of total CD45+ parenchymal cells. (D) Quantification of monocyte subtypes based on Ly6C expression represented as proportion of total CD45+ parenchymal cells (left) of total monocytes (right). (E) Quantification of macrophage subtypes identified as alveolar, interstitial, and transitional macrophages represented as proportion of total CD45+ parenchymal cells. (F) Quantification of classical dendritic cells and monocyte-derived dendritic cells represented as proportion of total CD45+ parenchymal cells. Group names and color are as follows: uninfected/mock, grey; low-dose vaccinated/0.5 μg S-2P, light red; high-dose vaccinated/5 µg S-2P, light green. Data are represented by the mean + /- the standard error of the mean. Statistical significance was determined using an unpaired one-way ANOVA with Tukey’s multiple comparisons test, and P values are represented above the bar graphs as follows: *, P < 0.05. (TIF) [file ppat.1013752.s005.tif]

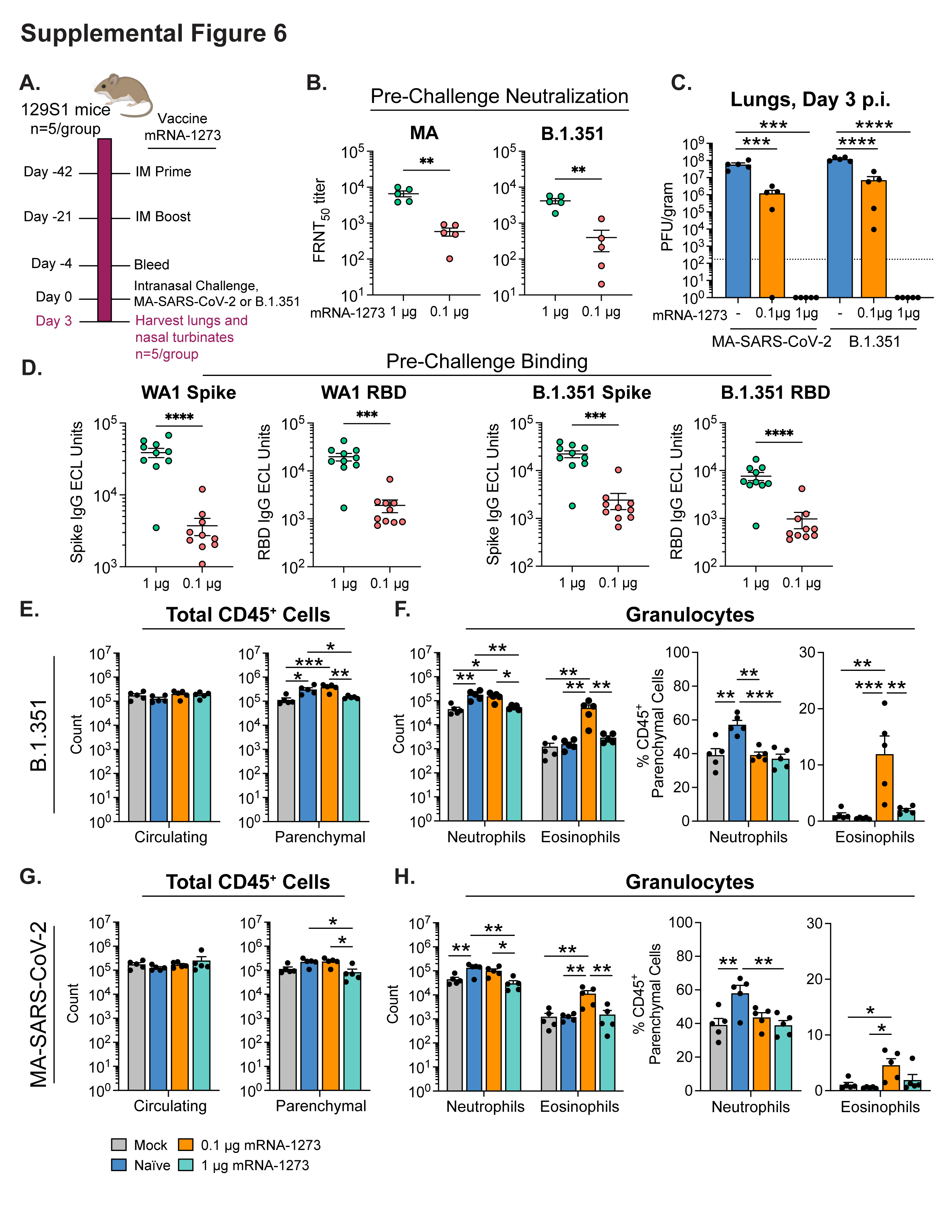

Supplement: S6 Fig — (A) Experimental schematic detailing vaccination of 129S1/SvImJ mice with low (0.1 µg) and high (1 µg) doses of mRNA-1273 and subsequent challenge with B.1.351 or mouse-adapted SARS-CoV-2 (MA-SARS-CoV-2). (B) Live virus FRNT50 titers against MA-SARS-CoV-2 and B.1.351 from serum collected 7 days before challenge. (C) Viral loads in the lungs as measured by plaque assay on day 3 p.i. (D) Serum IgG binding antibodies specific for WA1 and B.1.351 spike and RBD measured 7 days before challenge using a MesoScale Discovery V-Plex SARS-CoV-2 kit. (E-F) Quantification of lung immune cells by flow cytometry on day 3 p.i with B.1.351. (E) Absolute counts of CD45+ circulating cells (left) and absolute counts of CD45+ lung-resident parenchymal cells (right). (F) Quantification of parenchymal granulocytes identified as neutrophils and eosinophils represented as absolute counts (left) and proportion of total CD45+ parenchymal cells (right). (G-H) Quantification of lung immune cells by flow cytometry on day 3 p.i with MA-SARS-CoV-2. (G) Absolute counts of CD45+ circulating cells (left) and absolute counts of CD45+ lung-resident parenchymal cells (right). (G) Quantification of parenchymal granulocytes identified as neutrophils and eosinophils represented as absolute counts (left) and proportion of total CD45+ parenchymal cells (right). Group names and color are as follows: uninfected/mock, grey; naïve infected/SARS-CoV-2, blue; low-dose vaccinated infected/0.5 μg S-2P, SARS-CoV-2, orange; high-dose vaccinated infected/5 µg S-2P, SARS-CoV-2, teal. Data are represented by the mean + /- the standard error of the mean. Statistical significance was determined using an unpaired one-way ANOVA with Tukey’s multiple comparisons test, and P values are represented above the bar graphs as follows: *, P < 0.05; **, P < 0.01; ***, P < 0.001; ****, P < 0.0001. Created in BioRender. Suthar, M. (2025) https://BioRender.com/flyeqcf. (TIF) [file ppat.1013752.s006.tif]

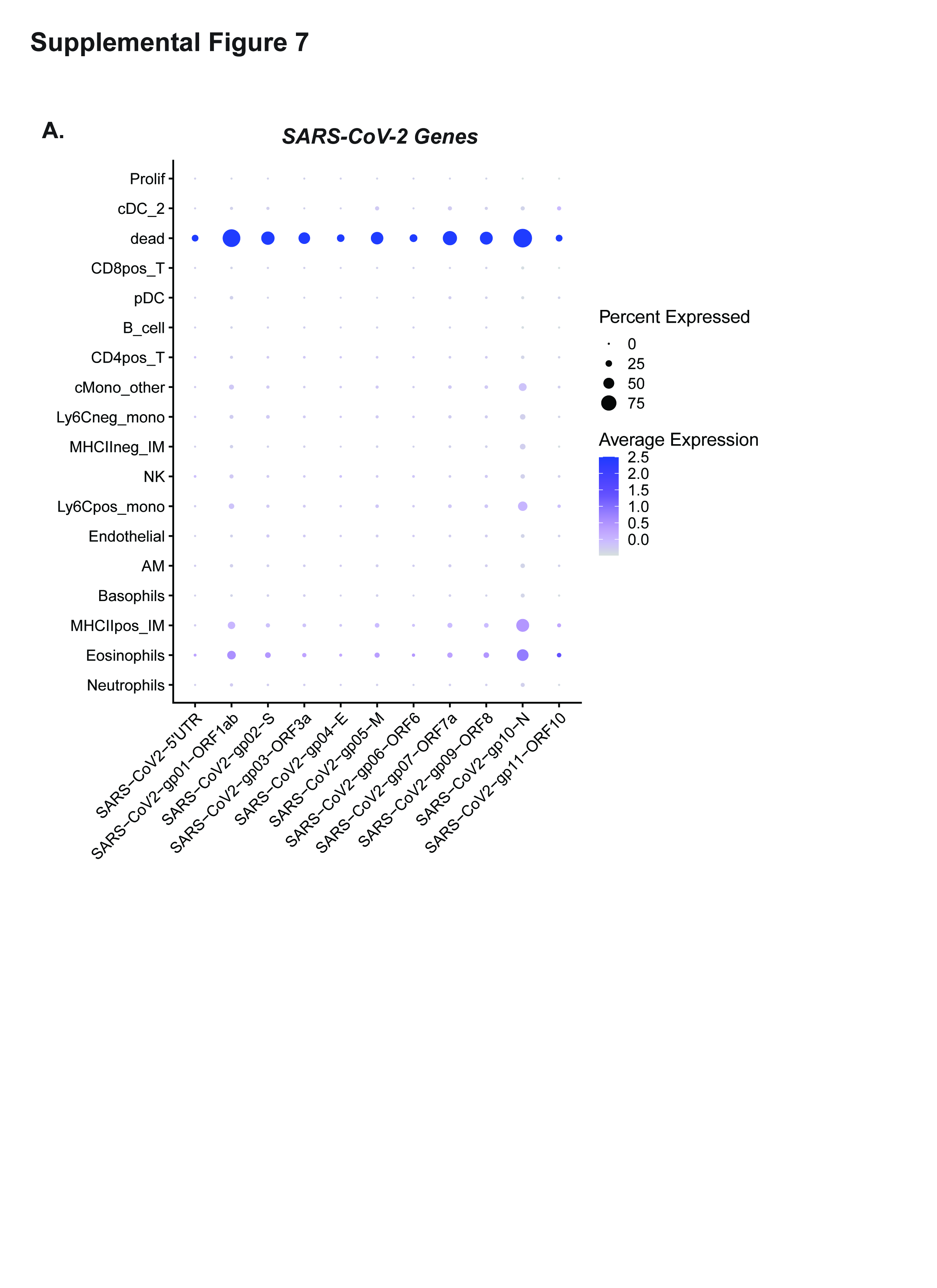

Supplement: S7 Fig — On day 3 p.i. lung parenchymal cells were isolated and evaluated by scRNA-seq. The average expression and percent of cells expressing each SARS-CoV-2 gene detected was determined for each cell type. These data are represented as a bubble plot, where the size of the circle represents the percent of cells expressing the given gene and the color of the fill represents the average expression among those cells. (TIF) [file ppat.1013752.s007.tif]

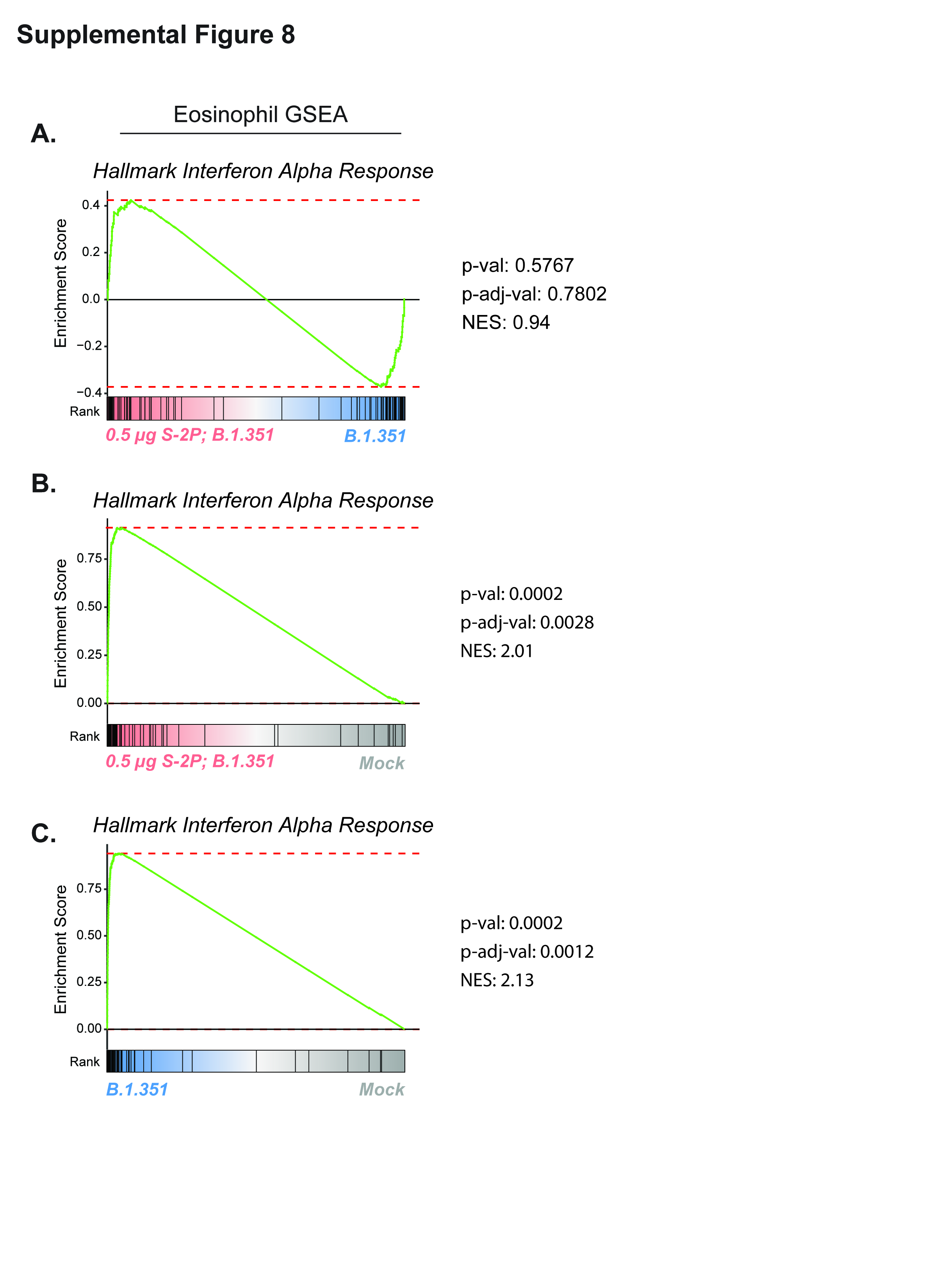

Supplement: S8 Fig — GSEA analysis plots for group pairs. (A) 0.5 μg S-2P-vaccinated infected vs naïve infected, (B) 0.5 μg S-2P-vaccinated infected vs mock, (C) naïve infected vs mock. (TIF) [file ppat.1013752.s008.tif]

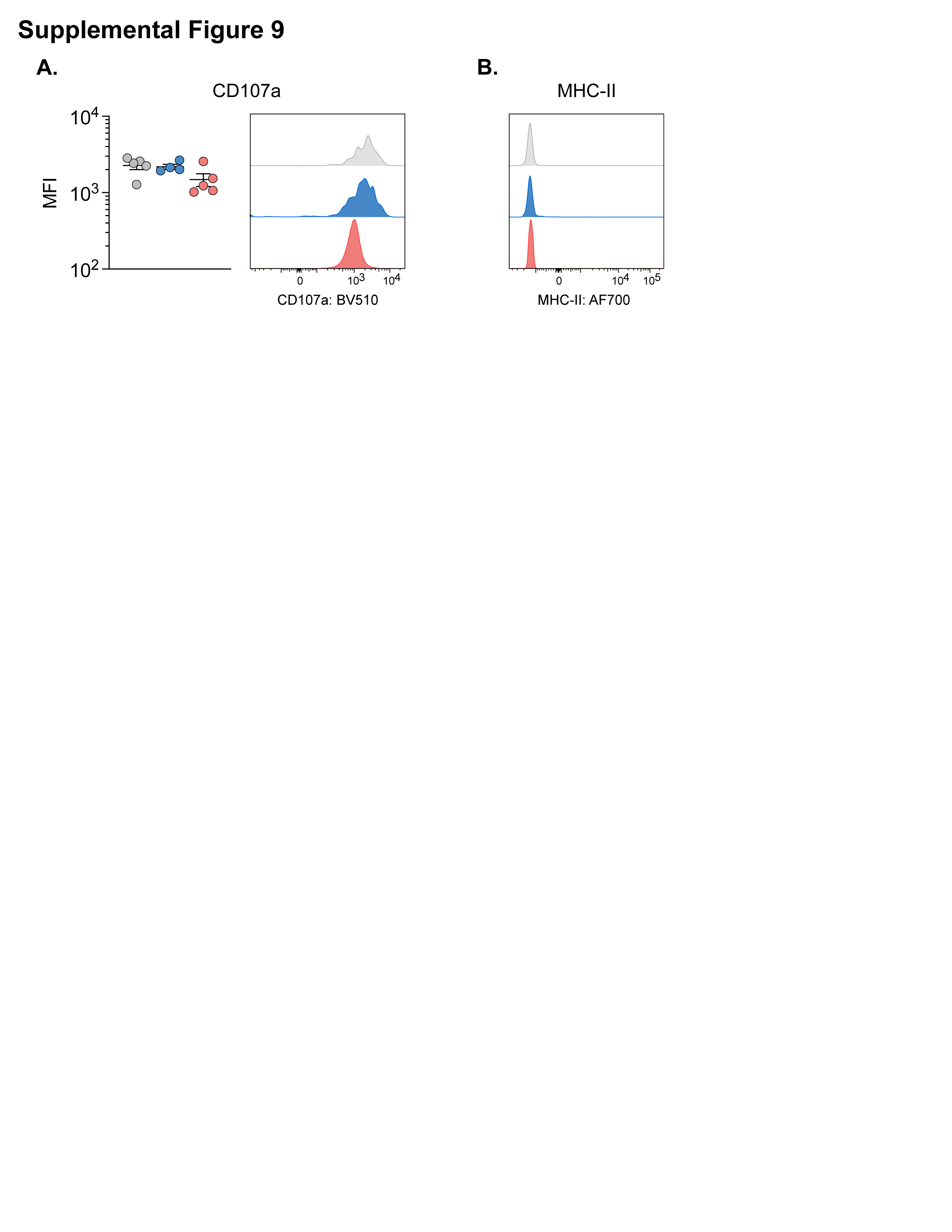

Supplement: S9 Fig — (A) Plots displaying mean fluorescence intensity (MFI). (B) Representative histograms of expression for each group. Group names and color are as follows: uninfected/mock, grey; naïve infected/SARS-CoV-2, blue; low-dose vaccinated infected/0.5 μg S-2P, SARS-CoV-2, red. (TIF) [file ppat.1013752.s009.tif]
